# What the papers say

**DOI:** 10.1093/jhps/hnaf034

**Published:** 2025-08-04

**Authors:** Ali Bajwa

**Affiliations:** Villar Bajwa Practice, The Princess Grace Hospital, London, W1G 6PU, United Kingdom

## Abstract

The *Journal of Hip Preservation Surgery* (*JHPS*) is not the only place where work in the field of hip preservation can be published. Although our aim is to offer the best of the best, we are continually fascinated by work, which finds its way into journals other than our own. There is much to learn from it, and so *JHPS* has selected six recent and topical subjects for those who seek a summary of what is taking place in our ever-fascinating world of hip preservation. What you see here are the mildly edited abstracts of the original articles, to give them what *JHPS* hopes is a more readable feel. If you are pushed for time, what follows should take you no more than 10 min to read. So here goes …

## Global acetabular retroversion is associated with increased conversion to total hip arthroscopy after primary hip arthroscopy: a propensity-matched analysis with minimum 8-year follow-up

The purpose of this study by Lee *et al*. [1] was to compare long-term survivorship following primary hip arthroscopy between patients with global acetabular retroversion and a propensity-matched control group. This retrospective study queried patients >18 years, with preoperative hip and pelvic radiographs and a minimum 8-year follow-up that underwent hip arthroscopy by a single surgeon for the treatment of symptomatic labral tears secondary to femoroacetabular impingement (FAI). Patients with global acetabular retroversion, as indicated by the presence of a crossover sign, ischial spine sign, and posterior wall sign on preoperative pelvic radiographs, were propensity-score matched 1:1 by age, sex, body mass index (BMI), and labral treatment (repair *versus* debridement) to controls. Baseline demographic, radiographic, and intraoperative variables were compared between cohorts. Cox multivariate regression controlling for global retroversion and Tönnis grade was used to assess conversion to total hip arthroplasty (THA). Patient-reported outcome measures (PROMs) were compared between cohorts.

Overall, 49 patients with global retroversion were 1:1 matched to 49 controls, with mean follow-up of 10.7 ± 2.1 years and 11.1 ± 2.8 years, respectively. There were no significant differences in baseline demographics, radiographic findings, or intraoperative findings between cohorts. Unadjusted Kaplan–Meier survival curves analysed by log-rank test demonstrated a significantly decreased survivorship among patients with global retroversion (68.6%) compared to controls (83.9%) at final follow-up. Cox multivariable regression controlling for Tönnis grade demonstrated that patients with global retroversion had a significant greater risk of THA conversion (hazard ratio, 3.94). There were no differences in PROMs at the final follow-up between cohorts.

The authors thus concluded that the patients with global acetabular retroversion had significantly inferior THA-free survivorship at minimum 8-year follow-up relative to matched controls, despite no differences in PROMs at final follow-up for patients who did not undergo THA. These findings suggest that global retroversion by preoperative radiographic assessment may be a useful predictor of long-term failure and that patients should be counselled accordingly.

## Hip arthroscopy patients experiencing greater neighbourhood-level socioeconomic disadvantage are more likely to achieve minimal clinically important differences in functional outcomes at 1-year follow-up

In this study, Gillinov *et al*. [2] investigate the influence of neighbourhood-level socioeconomic status (SES) on functional outcomes following hip arthroscopy. In their retrospective analysis of prospectively collected data, they queried patients aged ≥18 years with a minimum 1-year follow-up who underwent hip arthroscopy for the treatment of symptomatic labral tears secondary to FAI. The study population was divided into ADI_Low_ and ADI_High_ cohorts according to Area Deprivation Index (ADI) score, a validated measurement of neighbourhood-level SES standardized to yield a score between 1 and 100. Collected PROMs included the modified Harris Hip Score, Nonarthritic Hip Score, Hip Outcome Score (HOS)-Activities of Daily Living (HOS-ADL), HOS-Sports Specific Subscale (HOS-SSS), 33-item International Hip Outcome Tool (iHOT-33), Visual Analogue Scale (VAS) pain score, and patient satisfaction.

A total of 228 patients met the inclusion criteria and were included in the final analysis. After stratifying patients by ADI score, the ADI_Low_ (*n* = 113; mean ADI: 5.8 ± 3.0; range: 1 to 12) and ADI_High_ (*n* = 115; mean ADI: 28.0 ± 14.5; range: 13 to 97) cohorts had no differences in baseline patient demographics. ADI_High_ patients reported significantly worse preoperative baseline scores for all five PROMs; however, these differences were not present by 1-year follow-up. Furthermore, patients in both cohorts achieved similar rates of Minimal Clinically Important Differnce (MCID) for all five PROMs and Patient Acceptable Symptom State (PASS) for four PROMs. When controlling for patient demographics, patients with higher ADI scores had greater odds of achieving MCID for all PROMs except for iHOT-33.

In conclusion, the authors noted that although hip arthroscopy patients experiencing greater neighbourhood-level socioeconomic disadvantage exhibited significantly lower preoperative baseline scores, this disparity resolved at 1-year follow-up. In fact, when adjusting for patient characteristics, including ADI score, more disadvantaged patients achieved greater odds of achieving MCID. The present study is merely a first step towards understanding health inequities among patients seeking orthopaedic care. Further development of clinical guidelines and health policy research are necessary to advance care for patients from disadvantaged communities.

## Good mid-term clinical outcomes and low arthroplasty conversion rates after hip arthroscopy with labral debridement without refixation or reconstruction

In this study, Gahleitner *et al*. [3] investigate the 5-year outcomes of hip arthroscopy for cam or pincer-type FAI and associated labral tears in a defined patient population.

Patients who underwent hip arthroscopy for cam or pincer-type arthroscopy FAI and labral tears at their hospital in the past 5 years were included. All patients who underwent revision procedure such as a THA, a subsequent hip arthroscopy at another hospital, or had primary osseous diseases were excluded. Patients were contacted *via* mail and asked to answer a clinical questionnaire called the ‘Hip Osteoarthritis Outcome Score’ (HOOS) and to indicate whether there was a second surgery like a subsequent arthroscopy or THA. Results: There were 77 hip arthroscopies in 75 patients the last 5 years. A total of 29 patients responded. Those who did not respond were contacted *via* phone. All in all, they obtained the results of 49 patients (50 hips; 29 right, 19 left, and 1 bilateral) who underwent hip arthroscopy over the past 5 years. The mean age at the time of operation was 41 years. Our results were as follows: 24 hips had an isolated labral tear, 49 hips had a combined FAI pathology with cam and/or pincer-type impingement and labral tears, 3 patients had a posttraumatic FAI, and 1 patient suffered from hip chondromatosis, who were subsequently excluded; further, 22 patients (23 procedures) were lost to follow-up. HOOS contains various subscales; only the postoperative result of subscale 1 (symptoms) did not show a statistically significant improvement compared with the preoperative value. All other subscales showed a statistically significant improvement in comparison with the preoperative condition. Five patients (10.2%) still experienced symptoms, so they performed a THA as a second surgical procedure. One patient was revised due to chondromatosis. One patient was revised at another centre, and another was excluded because of chondromatosis.

The authors suggest that the 5-year follow-up results of hip arthroscopy proved successful outcomes. Hip arthroscopy is an effective treatment for FAI in order to delay primary THA, regaining mobility and range of motion (ROM) and reducing pain. Longer-term studies with a larger cohort are necessary.

## Comparative outcomes of hip arthroscopy for FAI in football and non-football athletes: a clinical analysis

Riccobono *et al*. [4] note that FAI is a common cause of hip pain and dysfunction, particularly among athletes, including football players. The condition is characterized by abnormal contact between the femoral head–neck junction and the acetabulum, leading to cartilage damage and labral tears. Hip arthroscopy has emerged as a minimally invasive treatment option, offering faster recovery and improved outcomes compared to traditional surgery. This study aimed to compare outcomes between football players and non-football athletes undergoing hip arthroscopy for FAI.

This retrospective, single-centre study analysed a database of patients undergoing hip arthroscopy for FAI between 2007 and 2023. The study compared football players (*n* = 16) and non-football athletes (*n* = 16), matched for age, sex, and BMI. Preoperative assessment included the HOS, VAS, and other functional questionnaires. Radiographic evaluations included the alpha and Wiberg angles, and intraoperative findings were recorded. The surgical approach involved femoral and acetabular osteoplasty, labral repair, or labrectomy, depending on injury morphology.

Both groups showed similar preoperative pain levels (VAS) and functional scores. However, significant differences were observed in the Tegner and Hip Sports Activity Scores, with football players showing higher activity levels preoperatively. Both groups demonstrated significant improvements in alpha and Wiberg angles post-surgery. The surgery duration was similar between groups, and no significant differences in postoperative outcomes were found between football and non-football players.

The authors concluded that hip arthroscopy is effective for both football and non-football players with FAI, with both groups experiencing significant improvements in hip joint function and pain relief. While preoperative functional scores differed, particularly in activity levels, both groups benefited from similar postoperative outcomes, suggesting that the surgical approach is suitable for active individuals across different sports. Further research is needed to explore long-term outcomes and return-to-sport rates in these populations.

## The impact of hip arthroscopy on the progression of hip osteoarthritis in patients with FAI syndrome: a systematic review and meta-analysis

In this study, Lameire *et al*. [5] state that hip arthroscopy (HA) for the surgical management of FAI syndrome (FAIS) provides reliable improvements in pain and function; however, debate remains regarding the impact of HA on the progression of osteoarthritis (OA). Their aim was to determine whether HA for FAIS reduces the progression of OA and the risk of conversion to THA.

A systematic electronic search of articles in Medline, Embase, and ClinicalTrials.gov databases was performed under the PRISMA (Preferred Reporting Items for Systematic Reviews and Meta-Analyses) guidelines, with 5046 articles remaining after duplicates were removed. All papers addressing HA for FAIS that reported radiographic progression of hip OA with a follow-up of ≥2 years were eligible for inclusion. Studies assessing labral reconstruction, revision HA, case reports, studies with <10 patients, and patients with hip dysplasia or rheumatoid arthritis were excluded. A total of 322 studies progressed to full text, and 16 studies were ultimately included in this review. Studies were divided based on short-term (ST) (2 to <5 years), mid-term (MT) (5 to <10 years), and long-term (LT) (>10 years) follow-ups. A meta-analysis of homogenous studies and outcomes was performed; otherwise, descriptive statistics were presented.

Sixteen studies (2278 hips) with FAIS were included, in which 1196 hips underwent HA and 1082 hips were treated nonoperatively. There were eight ST studies, four MT studies, and four LT studies. A meta-analysis of two comparative studies found 32% less risk of progression of radiographic OA (any increase in grading) with HA compared with nonoperative management. In addition, there was a nonsignificant 23% decreased risk of conversion to THA/hip resurfacing with HA. For all studies, there was a progression of hip OA ranging from 0% to 37.1% for ST studies, 11.5% to 23% for MT studies, and 4.3% to 28% for LT studies.

The authors concluded that their systematic review demonstrated that studies of patients undergoing HA for FAIS demonstrated increased radiographic progression of hip OA over time. Although significantly limited by only two retrospective cohort studies, subgroup analysis comparing operative *versus* nonoperative management demonstrated a 32% reduction in the radiographic progression of OA (any increase in grading) at the LT follow-up. However, there were no significant differences in the risk of THA/hip resurfacing. Future long-term, high-level controlled studies are needed to help further understand this important clinical question.

## Three-dimensional lower limb kinematics and kinetics in FAIS patients with and without borderline developmental dysplasia of the hip during level walking

In this study, Hao *et al*. [6] state that the impact of FAIS on gait has been reported; however, no studies have documented the effects of borderline developmental dysplasia of the hip (BDDH) combined with FAIS on gait. This study aimed to evaluate the kinematic and kinetic abnormalities of the lower extremities in patients with combined FAIS and BDDH during level walking.

A total of 42 participants were included, consisting of 14 patients with FAIS + BDDH, 14 with isolated FAIS and 14 healthy controls. Full-cycle kinematic and kinetic data were collected *via* motion capture and force plates. Gait analysis was performed in three planes (sagittal, coronal and transverse) for the hip, knee, ankle and pelvis joints. The ROM, kinematics and kinetics were compared across the three groups.

Compared with isolated FAIS patients, FAIS + BDDH patients presented a significantly greater hip flexion angle during terminal stance. Moreover, the hip abduction moment was significantly reduced in the loading response and midstance phases in FAIS + BDDH patients. The knee extension moment was significantly reduced during terminal stance in both FAIS groups. The ankle dorsiflexion angle was significantly greater during midstance in FAIS + BDDH patients than in healthy controls, with concomitant reductions in the ankle dorsiflexion moment. No significant differences were found in the ROM of the pelvis or hip joints and hip moment arm among the three groups.

The authors thus concluded that compared with patients with isolated FAIS, patients with FAIS combined with BDDH exhibit a gait pattern characterized by biomechanical defects of the hip joint similar to DDH, increased knee stiffness, and compensatory alterations in the ankle joint.

Conflict of interest: None declared.
